# Labeling messages as AI-generated does not reduce their persuasive effects

**DOI:** 10.1093/pnasnexus/pgag008

**Published:** 2026-02-10

**Authors:** Isabel O Gallegos, Chen Shani, Weiyan Shi, Federico Bianchi, Izzy Gainsburg, Dan Jurafsky, Robb Willer

**Affiliations:** Department of Computer Science, Stanford University, Stanford, CA 94305, USA; Stanford Law School, Stanford University, Stanford, CA 94305, USA; Department of Computer Science, Stanford University, Stanford, CA 94305, USA; Department of Electrical and Computer Engineering and Khoury College of Computer Sciences, Northeastern University, Boston, MA 02115, USA; Department of Computer Science, Stanford University, Stanford, CA 94305, USA; Department of Sociology, Stanford University, Stanford, CA 94305, USA; Department of Computer Science, Stanford University, Stanford, CA 94305, USA; Department of Sociology, Stanford University, Stanford, CA 94305, USA

**Keywords:** persuasion, artificial intelligence, large language models, public policy, law and technology

## Abstract

As generative AI enables the creation and dissemination of information at massive scale and speed, it is increasingly important to understand how people perceive AI-generated content. One prominent policy proposal requires explicitly labeling AI-generated content to increase transparency and encourage critical thinking about the information, but prior research has not yet tested the effects of such labels. To address this gap, we conducted a survey experiment (N=1,601) on a diverse sample of Americans, presenting participants with an AI-generated message about several public policies (e.g. allowing colleges to pay student-athletes), randomly assigning whether participants were told the message was generated by (i) an expert AI model, (ii) a human policy expert, or (iii) no label. We found that messages were generally persuasive, influencing participants’ views of the policies by 9.74 percentage points on average. However, while 92.0% of participants assigned to the AI and human label conditions believed the authorship labels, labels had no significant effects on participants’ attitude change toward the policies, judgments of message accuracy, nor intentions to share the message with others. These patterns were robust across a variety of participant characteristics, including prior knowledge of the policy, prior experience with AI, political party, education level, and age. Given current levels of trust in AI content, these results imply that, while authorship labels would likely enhance transparency, they are unlikely to substantially affect the persuasiveness of the labeled content, highlighting the need for alternative strategies to address challenges posed by AI-generated information.

## Introduction

Generative AI can now write persuasive content at unprecedented scale and speed (1, 2). While AI applications to politics may be positive, reducing conspiracy beliefs and facilitating compromise (3), advancing capabilities raise concerns about negative applications, such as AI-powered influence operations, misinformation campaigns, and other deceptive political activities (e.g. (1, 4, 5)). For example, a small group could flood platforms with misleading AI-generated content, manipulating perceived public support and distorting democratic discourse. Complicating matters, people struggle to distinguish AI from human-written content (6). Thus, the prospect of a massive-scale influx of content produced by AI could undermine trust in our information ecosystem. One proposed policy response is to require that AI-generated content be identified with an authorship label (7). For instance, the European Union’s AI Act requires that those deploying AI-generated content provide AI labels. The AI Labeling Act and AI Disclosure Act of 2023 call for similar provisions. These beg a critical question: does the knowledge that content was generated by AI meaningfully influence its impact? Do AI labels shape the influence of AI-generated content on people’s views of politics and public policy?

Prior work has focused on either the persuasiveness of AI-generated content *without* AI labels (e.g. (1, 2, 4)), or *perceptions* of the credibility, reliability, or quality of labeled information (e.g. (8–10)). Importantly, prior research finds that ratings of the quality of political content are not necessarily associated with the content’s persuasiveness (11), meaning that persuasion is best studied directly by measuring its potential impact on individuals’ attitudes. Here, we advance the literature by investigating the *persuasiveness* of AI-generated persuasive messages on policy issues, and how AI labels impact that influence. There is good reason to expect that labeling AI-generated content could reduce its persuasive impact. Prior studies have found that people generally prefer human content over AI content in settings such as news (10), public health messaging (8), and social media content (9) due to perceptions that AI sources are less trustworthy or accurate than human ones. To test whether the preference for human- over AI-authored content persists in the context of persuasion, we investigate the hypothesis that content labeled as AI-generated will be less persuasive than that labeled as human-written. On the other hand, AI labels may also trigger perceptions of expertise that could lead humans to be as or more persuaded than human-generated content (12), an effect amplified by developers’ own announcements of advanced model capabilities. To explore this competing perspective, we also test an alternative hypothesis that content labeled as AI-generated will be more persuasive than unlabeled content.

We conducted a pre-registered (https://osf.io/r2eak) survey experiment testing the impact of different authorship labels on the influence of messages about public policies in four domains—geoengineering, drug importation, college athlete salaries, and social media platform liability—to establish the robustness of our findings across topical domain. Messages must be persuasive to assess the effects of authorship labels on the extent of their persuasiveness. Therefore, we selected lesser-discussed and nonpolarizing policy issues to maximize the likelihood that participants would be open to persuasion, given that weak prior attitudes enhance message receptivity. We present participants with an AI-generated message, with factual errors manually corrected, about one public policy and randomly assign whether participants are told they receive (i) *information generated by an **expert AI model** trained in U.S. policy*, (ii) *information written by a **policy expert** trained in U.S. policy*, or (iii) *information*. The expertise qualifier follows public communication about models like Claude (“undergraduate level expert knowledge,” “graduate level expert reasoning” (https://perma.cc/R78Z-LKXZ)) and GPT-4 (“advanced reasoning capabilities,” 99th-percentile exam performance (https://perma.cc/85ER-AKM3)). We control the content of the messages, experimentally varying only the authorship disclosure.

## Results

The intervention changed perceptions of whether the author was AI or human. 94.6% of participants assigned to the *AI label* condition and 89.3% of those in the *human label* condition believed the author was the same as their assigned condition. In the *no label* condition, 39% of participants believed the message was human-written, 31% believed it was AI-generated, and the remainder were unsure. The *no label* condition mirrors the common scenario of online content with no specified author. The *human label* condition offers a direct comparison to the *AI label* condition, highlighting differences between two explicitly identified sources. Consistent with earlier demonstrations of AI persuasive power, the messages, generated by AI, were persuasive, influencing participants’ views of the policies by 9.74 (SE=0.41) points on average on the 0–100 scale (1, 4, 5).

**Fig. 1.**
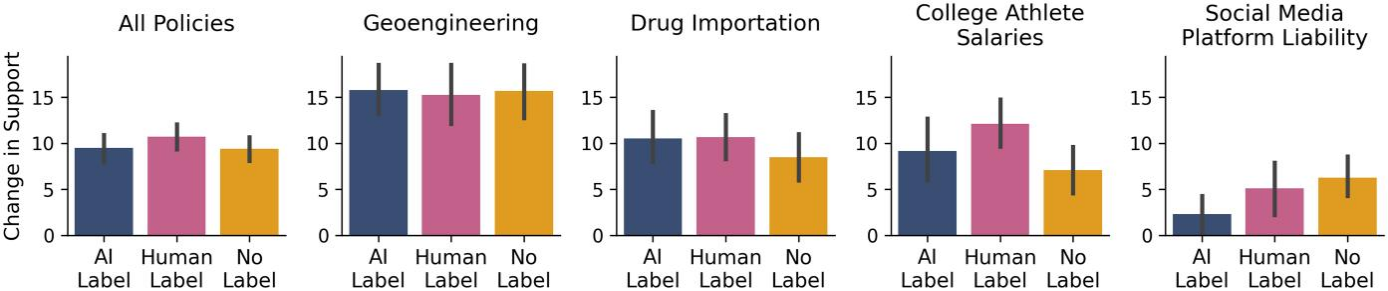
Mean and 95% CI for the difference between postintervention and preintervention support.

Across all policies, we find no evidence that AI labels significantly change the persuasiveness of the political messaging. Mean differences and 95% CI between postintervention and preintervention policy support measures for all policies are given in Fig. 1. We find that, even though participants assigned to the *human label* condition tended to increase their policy support slightly more than participants assigned to the *AI label* condition, these differences between conditions were not significant (b=−1.04, CI=[−2.89,0.80], P=0.27). Comparing the *AI label* condition and the *no label* condition, we again see no statistically significant difference (b=0.11, CI=[−1.73,1.96], P=0.91). We also ask participants for their confidence in their support, judgments of how accurate the message was, and intentions to share it with others. We do not observe significant differences between any pairs of the three labeling conditions for any of these dependent variables. For details, see https://osf.io/gqzf6.

Additionally, we pre-registered analyses to exclude participants who failed two manipulation check questions that checked if they believed the author was the same as their assigned condition. Even though 92.0% of participants assigned to the *AI* and *human label* conditions believed the authorship labels, this filtered analysis ensured that we retained only those participants who were successfully influenced by the manipulation (N=1,515). The number of participants excluded is comparable across the *AI label* (N=29) and *human label* (N=57) conditions; we retain all participants in the *no label* condition. These results converge with the prior results. Running the same analyses on the filtered data as conducted on the complete data, we find borderline to no significance in the difference between the *AI label* and *human label* conditions (b=−1.82, CI=[−3.74,0.10], P=0.063), nor between the *AI label* and *no label* conditions (b=−0.088, CI=[−1.96,1.78], P=0.92).

We also test whether any of five potentially impactful background characteristics of participants—political party identity, prior knowledge about the topic, prior experience with AI tools, education level, and age—significantly moderated the effects of AI labels on the persuasiveness of messages. For each moderator, we add an interaction term between each of the labeling condition dummy variables. We additionally perform subgroup analyses to explore these effects. While Americans on the whole may not respond negatively to AI labels, older individuals are more likely to react negatively to AI-labeled content compared to human-labeled content (b=−3.13, CI=[−5.80,−0.46], P=0.044, with Benjamini–Hochberg adjustment). However, when examining other characteristics, we find there are no effects in subgroups of interest. Thus, while labels may improve transparency, they do not necessarily reduce the persuasive impact of content, shedding light on the potential shortcomings of policy proposals that rely on AI labels alone to address challenges posed by AI-generated information.

## Discussion

We find that AI labels are unlikely to substantially affect the persuasiveness of labeled content. These findings are consistent with conversational studies, which find that AI interactions were persuasive, but did not differ significantly across labeling conditions (13–15). We note these results reflect the current state of AI capabilities and their public perceptions, which may evolve. In particular, AI-generated persuasive communications may be uniquely impervious to concerns about AI manipulation. For instance, in ours and similar studies (1, 3), the AI-generated messages were deliberately evidence-based and logical, minimizing hallucinations and relying on credible sources. Because fact-based communication tends to be persuasive regardless of its author, these messages may have been especially resistant to authorship labels. In the future, AI persuasion may evolve toward more personal or microtargeted styles. In such cases, labels could carry greater penalties, especially if AI mimics human-like appeals. Yet two caveats remain: such uses may be limited by labeling requirements, and current evidence suggests AI’s persuasive edge stems from its fact-based style (1, 3). Thus, the influence of AI labels will depend on how these systems are deployed and regulated.

An important limitation of these results is that they reflect how Americans engage with AI in 2024. We recruited participants from Prolific, where familiarity with AI-generated text is relatively high. This may enhance the relevance of our findings for near-future contexts with widespread AI exposure, while limiting generalizability to individuals unfamiliar with AI. Understandings of AI are likely to shift over time as people become more familiar with expanding capacities and limitations of AI models, which may change how people respond to content produced by AI vs. human authors. Responsible assessment of the impacts of these labeling policies will be best achieved through replicating the study in the future.

Next, though we do not find significant effects between labeling conditions and conducted an a priori power analysis, it is possible our sample size was insufficient to detect a small but real difference between labels. However, even small effects may be meaningful when scaled across large populations, especially in high-stakes contexts like elections or public health, or when such effects may propagate through social networks. Future research with larger samples may be necessary to assess such subtle but important shifts. We note that, to estimate the potential impact of an AI labeling policy, the most appropriate baseline for comparison is the *no label* condition, since this is how human-generated content would appear under most policy proposals; the effects of AI labels compared to the *no label* condition were clearly null.

This work is also likely overly simplistic, given theories that suggest that people leverage many heuristics beyond the source of information alone to make judgments. In particular, our “expert AI” and “policy expert” labels combine a source cue with a credibility-enhancing qualifier. Thus, our null effects may stem from perceptions of AI, perceptions of expertise, or their interaction. This framing reflects descriptions of current frontier models and is consistent across the *AI* and *human label* conditions. However, this limits generalizability to contexts in which AI is labeled more generically or skeptically. Future work can disentangle these factors by varying both the source type and the credibility signal. Indeed, one possibility is that expertise cues may carry more weight when attached to humans but be discounted for AI models. Besides our expertise qualifier, messages are stripped of credibility cues that often accompany real-world content, such as author identity, institutional affiliation, or platform. While this approach allows us to isolate the effect of authorship labels, our findings should be interpreted primarily as applying to decontextualized or unattributed messages, which may be common in generative AI applications, but are less representative of traditional institutional communication. Future research can also explore whether persuasion effects—especially for political content—differ when messages are attributed to specific AI models, as people may trust AI in general more or less than particular platforms if platforms develop partisan content or user bases.

## Materials and methods

The study features a 2 (time: pre- vs. post-intervention) × 4 (policy domain: geoengineering vs. drug importation vs. college athlete salaries vs. social media platform liability) × 3 (authorship label: AI label vs. human label vs. no label) within-between-between-subject design. We randomly sampled four policies from Ref. (2)’s Persuasion Dataset. We measure support, confidence, sharing intention, and accuracy judgment on 0 to 100 scales (1). We estimate regression models for each dependent variable, comparing (i) the *AI* and *no label* and (ii) the *AI* and *human label* conditions, and (iii) adding interaction terms for several possible moderators. We control for the policy and preintervention measure in each model. An a priori power analysis based on pilot data indicated we needed 125 participants per label-topic condition (1500 total) to detect a 3-point difference between AI and human labels on the 0–100 scale with 80% power at a significance level of 0.05. The study was approved by Stanford University’s Institutional Review Board. All participants provided and confirmed their informed consent. See Supplementary Information for full methods.

## Conclusions

In a time of rapidly evolving AI systems, policymakers have called for explicitly labeling AI-generated content. We have shown that the persuasive effect is nearly equivalent when information is labeled as generated by an expert AI model as when it is labeled as written by a policy expert or has no label. Thus, AI disclosure policies may only have a weak, or no, effect on people’s use of AI-labeled content. Beyond transparency, these policies serve other functions, such as helping users make informed decisions about content consumption or curbing the spread of AI-generated material, areas our study does not explore. At the same time, our results only capture perceptions in 2024 and of a general “expert AI” label, without other source cues. Nevertheless, our findings underscore the need for further research into how AI disclosure policies shape the information ecosystem.

## Supplementary Material

pgag008_Supplementary_Data

## Data Availability

The data underlying this article are available on the Open Science Framework (OSF) at https://osf.io/s97hz.

## Preprints

This manuscript was posted on a preprint: https://arxiv.org/abs/2504.09865.
