# Supplementary material for: Labeling messages as AI-generated does not reduce their persuasive effects
Source: PNAS Nexus. 2026 Feb 10;5(2):pgag008. doi: 10.1093/pnasnexus/pgag008 (PMC12887897; doi:10.1093/pnasnexus/pgag008)
Supplement: pgag008_Supplementary_Data [file pgag008_supplementary_data.pdf]

PNAS NEXUS

Supplementary Information for

Labeling messages as AI-generated does not reduce their persuasive effects

Isabel O. Gallegos, Chen Shani, Weiyan Shi, Federico Bianchi, Izzy Gainsburg, Dan Jurafsky, and Robb Willer

Corresponding Author: Isabel O. Gallegos
E-mail: iogalle@stanford.edu

This PDF file includes:

Supplementary text
SI References

Supplementary Information Text

1. Methods

Following the study design of (1), we measure persuasiveness by the change in policy support before and after reading a persuasive message. The study features a 2 (time: pre- vs. post-intervention) x 4 (policy domain: geoengineering vs. drug importation vs. college athlete salaries vs. social media platform liability) x 3 (authorship label: AI label vs. human label vs. no label) within-between-between-subject design. Participants read the exact same message about their assigned policy but are either told that it is (a) generated by an expert AI model trained in U.S. policy, (b) written by a policy expert trained in U.S. policy, or (c) given no authorship details. These labels align with proposed interventions to provide exogenous cues to signal the information's source, to help users evaluate the information (2), and the expertise qualifier aligns with public communication about models like Claude ("undergraduate level expert knowledge" and "graduate level expert reasoning" (https://perma.cc/R78Z-LKXZ)) and GPT-4 ("advanced reasoning capabilities," top 90th-percentile performance on the Uniform Bar Exam, and top 99th-percentile performance on the Biology Olympiad (https://perma.cc/85ER-AKM3)).

We randomly sampled four policy proposals from (3)'s Persuasion Dataset (CC BY-NC-SA 4.0), which contains a set of 56 claims about recently emerging issues, such as climate geoengineering research and collegiate athlete salaries. The dataset was explicitly constructed to contain less polarized issues, such that people are less likely to have well-established or deeply-held views in each policy domain. Thus, given the developing nature of the policies, the issues are lesser-discussed and non-polarizing, which can maximize the likelihood that participants would be open to persuasion, given that weak prior attitudes enhance message receptivity. This design choice increases the likelihood of detecting the effects of persuasive messages, compared to messages about highly polarized issues. By focusing on policies with weak priors, our experiment provides a liberal assessment—one that is as favorable as possible to identifying differences in persuasion effects across conditions.

In June 2024, we collected one message generated by OpenAI's GPT-4o model for each of the four policy proposals to persuade readers to support the policies. To control for persuasiveness, we employed techniques of evidence-based persuasion and expert endorsement described in (4)'s taxonomy. We manually edited the text only to correct any factual errors. For additional detail, see Section 2 of Supplementary Information.

We measure four dependent variables—support, confidence, sharing intention, and accuracy judgment—on 0 to 100 scales (1), with support pre-registered as the primary measure of persuasion. For the complete set of measures, see Section 3 of Supplementary Information. The study had four stages: (1) *pre-intervention stage*: participants were told they would participate in a public opinion survey; they then indicated their prior knowledge about the topic and their pre-intervention levels of support and confidence in their support; (2) *intervention stage*: participants read the persuasive appeal, with the message author identified as AI, human, or no label (randomly assigned); (3) *post-intervention stage*: participants indicated their post-intervention levels of support for the policies, confidence in their support, and also their judgments of how accurate the message was and their intentions to share it with others; and, (4) *post-study questionnaire*: this survey included an attention check item, questions measuring perceptions of the assigned message source, and demographic information. We then disclosed the study purpose and true authorship. The study was approved by Stanford University's Institutional Review Board. All participants provided and confirmed their informed consent.

We conducted an *a priori* power analysis based on pilot data to determine the required sample size. Assuming an effect size of 3 points on the 0 to 100 point scale for the support variable between the *AI label* and *human label* conditions, a sample size of 125 participants for each label-topic condition (1,500 participants in total) was required to achieve 80% power at a significance level of 0.05; this effect size is based on prior literature that has found very small effects between AI and human authorship disclosures along other perception measures (5–8). We recruited a total of 1,725 participants from Prolific between July 1-2, 2024. We excluded participants who failed the attention check, leaving 1,601 remaining. For one analysis, we also excluded participants who failed the manipulation check, with 1,515 remaining. For participant details, see Section 4 of Supplementary Information.

We estimate several regression models, following (1), to model multiple control variables and interaction effects. Persuasiveness is measured by the change in policy support from pre- to post-intervention. First, we regress post-intervention support on dummy-coded variables for the *AI label* and *no label* conditions (contrasted with *human label*) while controlling for the policy and pre-intervention support. We consider all policies within a single regression model following (9)'s recommendation and (1)'s methodology to improve causal inference compared to a single policy. Second, we again regress post-intervention support on dummy-coded variables for the *AI label* and *human label* conditions (contrasted with *no label*), again controlling for the policy and pre-intervention support. We repeat these regression analyses for confidence, accuracy, and sharing.

The code repository to generate all results is available at https://github.com/i-gallegos/ai-authorship-persuasion. Data is available at https://osf.io/s97hz.

2. Information Content

We use the following prompt to generate the information content from GPT-4o, employing techniques of evidence-based persuasion and expert endorsement described in (4)'s taxonomy to control for persuasiveness:

```
Persuade a reader to agree with the following proposal: "[POLICY]". To do so, write a persuasive statement of
100 words that contains two TRUE domain expert sources of TRUE empirical data, statistics, or facts in support of
the proposal, as well as one TRUE quotation from a TRUE domain expert.
```

The generated information contents, with factual errors manually corrected, are shown below:

> ***Geoengineering poses too many risks and should not be considered.***
> Geoengineering poses too many risks and should not be considered. According to several scientific studies, large-scale geoengineering could deplete ozone in the atmosphere, damage ecosystems, and even accelerate global warming. Additionally, a report from the Intergovernmental Panel on Climate Change (IPCC) notes that the consequences cannot yet be anticipated as the level of scientific understanding about geoengineering is low. Dr. Alan Robock, a renowned climate scientist, warns, "Scientists cannot possibly account for all of the complex climate interactions or predict all of the impacts of geoengineering. With so much at stake, there is reason to worry about what we don't know." These credible sources underscore the significant dangers associated with geoengineering, making it an unviable solution.

> ***Drug importation jeopardizes safety controls and the domestic pharma industry.***
> Drug importation jeopardizes safety controls and the domestic pharma industry. According to the Food and Drug Administration (FDA), 72% of active pharmaceutical ingredients are sourced from abroad, raising concerns about quality control and safety. A study by the National Association of Boards of Pharmacy found that the sale of counterfeit drugs used for chronic conditions, health maintenance, and infections is lucrative, generating over $200 billion a year in profit for global criminal networks. Dr. Janet Woodcock, Director of the FDA's Center for Drug Evaluation and Research, warns that the "use of foreign-sourced materials creates vulnerabilities in the U.S. drug supply." Protecting our domestic pharma industry is crucial for maintaining rigorous safety standards.

> ***College athletes should be paid salaries.***
> College athletes should be paid salaries because they generate significant revenue for their institutions. According to the NCAA, college sports brought in $18.9 billion in 2019 alone. Furthermore, a study conducted by Drexel University and the National College Players Association found that the annual fair market value of an average college football player is $178,000. As Jay Bilas, a former Duke basketball player and ESPN analyst, stated, "In my view, there is no legitimate justification to limit the compensation of an athlete, just as there is no legitimate justification to limit the compensation of coaches, administrators, or staff." Paying college athletes would ensure fair compensation for their contributions and help address the financial disparities they face.

> ***Social media platforms should be liable for harmful content posted by users.***
> Social media platforms should be liable for harmful content posted by users. According to a 2021 Pew Research Center study, 64% of Americans believe social media has a mostly negative effect on the way things are going in the country today. Additionally, a 2020 report by the Anti-Defamation League found that 44% of Americans experienced online harassment, with 77% of Americans wanting companies to make it easier to report hateful content and behavior. As cybersecurity expert Bruce Schneier states, "The biggest mistake we made with social media was leaving it as an unregulated space. Even now - after all the studies and revelations of social media's negative effects - social media in the US remains largely an unregulated 'weapon of mass destruction.'"

3. Measures

In the following survey questions, [*Policy Proposal*] denotes one of the policy proposals listed in Section 2, while [*Topic*] denotes one of the following policy domains, based on the participants' assigned conditions: *geoengineering*, *drug importation*, *college athlete salaries*, or *social media platform liability*.

A. Pre-Intervention.

1. How knowledgeable do you feel about [*Topic*]?
   - I am an expert on this topic.
   - I am very knowledgeable about this topic.
   - I am moderately knowledgeable about this topic.
   - I am slightly knowledgeable about this topic.
   - I have little to no knowledge about this topic.
2. Please indicate your level of agreement with the following statement: [*Policy Proposal*].
   - 0 = Strongly disagree
   - 50 = Neither agree nor disagree
   - 100 = Strongly agree
3. How confident are you in your responses?
   - 0 = Very unconfident
   - 50 = Neither confident nor unconfident
   - 100 = Very confident

B. Intervention. In the *AI label* condition, participants receive the following:

1. You will now receive information generated by an expert AI model trained in U.S. policy. We have collected a set of opinions about the topic from multiple expert AI models. Select the AI model that will provide the perspective you will read. (Participants select a number from 1 to 10.)
2. Consider the following information provided by expert AI model #[*Selection*]. [*Information Content*]

☐ I have read this information carefully.

In the *human label* condition, participants receive the following:

1. You will now receive information written by a policy expert trained in U.S. policy. We have collected a set of opinions about the topic from multiple policy experts. Select the person who will provide the perspective you will read. (Participants select a number from 1 to 10.)
2. Consider the following information provided by policy expert #[*Selection*]. [*Information Content*]

   ☐ I have read this information carefully.

In the *no label* condition, participants receive the following:

1. You will now receive information. We have collected a set of opinions about the topic. Select the message that you will read. (Participants select a number from 1 to 10.)
2. Consider the following information provided by Message #[*Selection*]. [*Information Content*]

   ☐ I have read this information carefully.

We include the participant article selection step to create the impression that participants could have received a range of viewpoints, rather than only a single persuasive message. Our goal was to reduce potential demand effects by suggesting that the information presented was not predetermined or ideologically one-sided. While this step introduces a minor element of agency, participants were not given any additional details about the information source beyond their assigned label, and the content they received was unaffected by which article they selected. Additionally, incorporating this small degree of choice mirrors how people often exercise agency in real-world information environments, such as choosing between AI models or news outlets, even if participants' selections here did not change the content they received. Thus, the procedure functions primarily as a randomization illusion designed to enhance credibility and neutrality without affecting the experimental manipulation.

C. Post-Intervention. After reading the information, participants respond to the following:

1. Please indicate your level of agreement with the following statement: [*Policy Proposal*]. You previously selected [*Pre-Intervention Selection*].
   - 0 = Strongly disagree
   - 50 = Neither agree nor disagree
   - 100 = Strongly agree
2. How confident are you in your responses? You previously selected [*Pre-Intervention Selection*].
   - 0 = Very unconfident
   - 50 = Neither confident nor unconfident
   - 100 = Very confident
3. How likely would you be to share this information with others if the topic came up (for example, in conversation or on social media)?
   - 0 = Very unlikely
   - 50 = Neither likely nor unlikely
   - 100 = Very likely
4. To the best of your knowledge, is the information accurate?
   - 0 = Very inaccurate
   - 50 = Neither accurate nor inaccurate
   - 100 = Very accurate

D. Post-Study Questionnaire. Participants pass the attention check if they select the first and third answers:

> People get their news from a variety of sources, and in today's world reliance on online news sources is increasingly common. We also want to know if people are paying attention to the question. To show that you've read this much, please ignore the question and select both "online sources only" and "about half online sources" as your two answers.
>
> About how much of your news consumption comes from online sources? Please include print newspapers that you read online (e.g., washingtonpost.com) as online sources.
>
> - Online sources only
> - Mostly online sources
> - About half online sources
> - Mostly television or print news, with some online sources
> - Television or print news only

We then ask about participants' perceptions of the information source, which includes two manipulation check questions (2 and 3, listed below). Participants passed the manipulation check if: (a) they were assigned to the *AI label* or *human label* condition and responded that the author "possibly" or "definitely" matched the assigned condition, *or* (b) if they reported not questioning the author's identity before being asked, *or* (c) if they were in the *no label* condition (where no author expectation was set).

> Thank you for providing your perspective on the policy proposals. We would now like to understand your experience and interactions with the information. Click the button to continue to a short questionnaire about your experience.

The following questions are asked only for survey completeness to understand the clarity, relevance, tone, and other qualities of the information you read.

1. (Optional) How, if at all, did the information or its author affect your opinions about the policy?
2. Do you think that this information was written by a human, or do you think that it was generated by an artificial intelligence (AI) model?
   - Definitely human-written
   - Possibly human-written
   - Not sure
   - Possibly AI-generated
   - Definitely AI-generated
3. Did you question whether this information was generated by a human or AI before we specifically asked you this?
   - Yes
   - No
4. Please rate your prior experience with conversational artificial intelligence (AI) models or large language model (LLM) tools. Examples include: ChatGPT (OpenAI), Claude (Anthropic), Gemini (Google), and Bing AI or Copilot (Microsoft).
   - I use conversational AI or LLMs more than once a week.
   - I use conversational AI or LLMs about once a week.
   - I use conversational AI or LLMs about once a month.
   - I use conversational AI or LLMs less than once a month.
   - I never use conversational AI or LLMs.
   - I have never heard of conversational AI or LLMs.

Finally, we ask participants about their demographic information:

1. What is the highest level of school you have completed or the highest degree you have received?
   - No formal schooling
   - Did not receive high school diploma
   - High school graduate
   - GED or equivalent
   - Some college
   - 2-year degree (e.g., associate degree)
   - Bachelor's degree
   - Master's degree
   - Professional or academic doctorate degree
   - Prefer not to answer
2. Please choose whichever race and/or ethnicity that you identify with (you may choose more than one option):
   - American Indian or Alaska Native
   - Asian
   - Black or African American
   - Hispanic or Latino
   - Middle Eastern or North African
   - Native Hawaiian or Pacific Islander
   - White
   - Other
3. Generally speaking, do you usually think of yourself as a Republican, a Democrat, an Independent, or other?
   - Republican
   - Democrat
   - Independent
   - I don't identify with any political party
   - Other
   - Prefer not to answer

Participants' age and gender are provided directly by Prolific.

4. Participants

We required participants to be English-speaking, U.S. residents, with an approval rate of 97-100 and at least 100 prior submissions on Prolific. We paid participants at a rate of $15.00 per hour. We recruited 1,725 participants to the study. An additional 39 participants started, were randomly assigned to a condition, but did not complete the study, resulting in an attrition rate of 2.21%. To assess whether this attrition varied systematically across experimental conditions, we estimated a logistic regression model predicting retention from dummy-coded variables for the *human label* and *no label* conditions (contrasted with *AI label*) while controlling for the policy. Retention in the *AI label* condition was not significantly different

from the *human label* condition (b=0.17, CI=[-0.64, 1.00], p=0.68), nor from the *no label* condition (b=-0.15, CI=[-0.92, 0.61], p=0.70). Thus, attrition is unlikely to have introduced bias into the experimental results we report. For detailed regression results, see https://osf.io/gqzf6.

It is possible that the persuasive effect may differ between participants who "definitely" believed the label matched their expectations about the author's identity and those who only "possibly" believed this. When we set the threshold for the manipulation check to only include participants who "definitely" believed the author was the same as their assigned condition (N=1417), the results still converge with prior results, with borderline to no significance, both when comparing the *AI label* to *human label* condition (b=-1.96, CI=[-3.99, 0.06], p=0.058), and when comparing the *AI label* to *no label* condition (b=-0.41, CI=[-2.36, 1.55], p=0.69). These results suggest that, whether participants have a strong or weak alignment between their assigned condition and their belief about the author's identity, the observed effects remain non-significant. We also investigate the effect on participants who failed the manipulation check to understand whether someone's belief that they are being tricked interferes with persuasion. There were only 86 participants who failed the manipulation check (N=29 in the *AI label* condition and N=57 in the *human label* condition). Because no participants in the *no label* condition could fail the manipulation check by definition, we compare only the *AI label* and *human label* conditions. We see a significant effect (b=6.90, CI=[0.11, 13.69], p=0.046) that participants in the *AI label* condition increase their support for the policy more than those in the *human label* condition. Because this group is very small and may not be representative, the result should be interpreted cautiously. That said, this may hint at a counterintuitive phenomenon worth future study: that suspicion or misattribution might modulate persuasion differently, perhaps leading those who sense they are being misled to engage more deeply or react more strongly to AI-labeled content.

Participant demographics are as follows:

Age: Mean=39.9, SE=0.31

Gender:

- Male: 46.6%
- Female: 53.2%

Race/Ethnicity:

- White: 67.5%
- Black or African American: 13.1%
- Asian: 7.6%
- Hispanic or Latino: 4.5%

Political Party:

- Democrat: 49.0%
- Independent: 24.7%
- Republican: 20.1%
- Does not identify with any political party: 4.7%

Education:

- Bachelor's degree or more: 57.9%
- Some college: 30.0%
- High school graduate: 10.4%
- GED or equivalent: 1.1%
- Did not receive high school diploma: 0.3%

AI Experience:

- I use conversational AI or LLMs more than once a week: 19.1%
- I use conversational AI or LLMs about once a week: 20.0%
- I use conversational AI or LLMs about once a month: 15.2%
- I use conversational AI or LLMs less than once a month: 27.0%
- I never use conversational AI or LLMs: 17.3%
- I have never heard of conversational AI or LLMs: 1.4%

References

1. H Bai, JG Voelkel, S Muldowney, JC Eichstaedt, R Willer, LLM-generated messages can persuade humans on policy issues. *Nat. Commun.* **16**, 6037 (2025).
2. S Feuerriegel, et al., Research can help to tackle AI-generated disinformation. *Nat. Hum. Behav.* **7**, 1818–1821 (2023).
3. E Durmus, et al., Measuring the persuasiveness of language models (2024).
4. Y Zeng, et al., How Johnny can persuade LLMs to jailbreak them: Rethinking persuasion to challenge AI safety by humanizing LLMs (2024).
5. E Karinshak, SX Liu, JS Park, JT Hancock, Working with AI to persuade: Examining a large language model's ability to generate pro-vaccination messages. *Proc. ACM on Human-Computer Interact.* **7** (2023).

6. C Longoni, A Fradkin, L Cian, G Pennycook, News from generative artificial intelligence is believed less in *Proceedings of the 2022 ACM Conference on Fairness, Accountability, and Transparency*, FAccT '22. (Association for Computing Machinery, New York, NY, USA), p. 97–106 (2022).
7. I Rae, The effects of perceived AI use on content perceptions in *Proceedings of the CHI Conference on Human Factors in Computing Systems*, CHI '24. (Association for Computing Machinery, New York, NY, USA), (2024).
8. B Toff, FM Simon, "Or they could just not use it?": The dilemma of AI disclosure for audience trust in news. *The Int. J. Press.* p. 19401612241308697 (2024).
9. C Fong, J Grimmer, Causal inference with latent treatments. *Am. J. Polit. Sci.* **67**, 374–389 (2023).
